# Acrylamide-Derived Ionome, Metabolic, and Cell Cycle Alterations Are Alleviated by Ascorbic Acid in the Fission Yeast

**DOI:** 10.3390/molecules27134307

**Published:** 2022-07-05

**Authors:** Marek Kovár, Alica Navrátilová, Renata Kolláthová, Anna Trakovická, Miroslava Požgajová

**Affiliations:** 1 Institute of Plant and Environmental Science, Faculty of Agrobiology and Food Resources, Slovak University of Agriculture in Nitra, Tr. A. Hlinku 2, 94976 Nitra, Slovakia; marek.kovar@uniag.sk; 2 Institute of Nutrition and Genomics, Faculty of Agrobiology and Food Resources, Slovak University of Agriculture in Nitra, Tr. A. Hlinku 2, 94976 Nitra, Slovakia; alica.navratilova@uniag.sk (A.N.); anna.trakovicka@uniag.sk (A.T.); 3 Institute of Animal Husbandry, Faculty of Agrobiology and Food Resources, Slovak University of Agriculture in Nitra, Tr. A. Hlinku 2, 94976 Nitra, Slovakia; renata.kollathova@uniag.sk; 4 AgroBioTech Research Center, Slovak University of Agriculture in Nitra, Tr. A. Hlinku 2, 94976 Nitra, Slovakia

**Keywords:** acrylamide, antioxidant capacity, ascorbic acid, cell cycle, ionome, morphology, oxidative stress, *Schizosaccharomyces pombe*

## Abstract

Acrylamide (AA), is a chemical with multiple industrial applications, however, it can be found in foods that are rich in carbohydrates. Due to its genotoxic and cytotoxic effects, AA has been classified as a potential carcinogen. With the use of spectrophotometry, ICP-OES, fluorescence spectroscopy, and microscopy cell growth, metabolic activity, apoptosis, ROS production, MDA formation, CAT and SOD activity, ionome balance, and chromosome segregation were determined in *Schizosaccharomyces pombe*. AA caused growth and metabolic activity retardation, enhanced ROS and MDA production, and modulated antioxidant enzyme activity. This led to damage to the cell homeostasis due to ionome balance disruption. Moreover, AA-induced oxidative stress caused alterations in the cell cycle regulation resulting in chromosome segregation errors, as 4.07% of cells displayed sister chromatid non-disjunction during mitosis. Ascorbic acid (AsA, Vitamin C), a strong natural antioxidant, was used to alleviate the negative impact of AA. Cell pre-treatment with AsA significantly improved AA impaired growth, and antioxidant capacity, and supported ionome balance maintenance mainly due to the promotion of calcium uptake. Chromosome missegregation was reduced to 1.79% (44% improvement) by AsA pre-incubation. Results of our multiapproach analyses suggest that AA-induced oxidative stress is the major cause of alteration to cell homeostasis and cell cycle regulation.

## 1. Introduction

Acrylamide (AA) is a monomeric substrate with the chemical formula C_3_H_5_NO and a molecular weight of 71.08. It is a synthetic, water-soluble, colorless, and odorless substance [1] widely used as an industrial chemical in the textile, plastic, and paper industries [2,3]. Acrylamide was discovered in foods in 2002 as a product of the so-called Maillard reaction between asparagine residues and reducing sugars at high temperature (above 120 °C), hence fried and baked starch-rich foods such as potato chips, French fries, crackers, cookies, coffee, and bread are the main sources of the accidental AA ingestion [4]. Intake of food-derived AA is estimated to be 0.3–2.0 µg kg^−1^ b.w. (body weight) in the human population [5]. The toxicology of AA has gained attention as its toxic spectrum was found to be wider than expected. AA was classified as a potential carcinogen by the International Agency for Research on Cancer (IARC) as its major metabolite glycidamide triggers neurotoxicity, genotoxicity, and reproductive toxicity in animals and humans due to its ability to bind DNA [6,7,8,9]. Intracellularly, AA non-enzymatically or by glutathione-S-transferases conjugates with glutathione (GSH) resulting in the formation of N-acetyl-S-(3-amino-3-oxypropyl)-cysteine. This in turn leads to depletion of the cellular GSH, thus triggering elevation of reactive oxygen species (ROS) formation leading to oxidative stress. Additionally, enhanced sensitivity to AA of cells depleted from Cu, Zn-superoxide dismutase (Sod1p), and AA-mediated decrease in catalase (CAT) expression and increase in malondialdehyde (MDA) formation, confirmed the oxidative activity of AA [10,11,12]. Due to increasing evidence of AA toxicity, the need to understand the mechanism of AA activity and the prophylactic/protective therapy’s capability to reduce or minimize AA-induced alterations has risen. It has been shown that compounds with antioxidant properties such as curcumin, β-carotene, vanillin, caffeic acid, rosmarinic acid, Vitamin E, or 5-amino salicylic acid have the ability to inhibit AA-triggered cytotoxicity through their direct effect on ROS scavenging activity [5,13,14]. In addition, the amount of AA in the food colorant ammonia caramel that is produced by Maillard reaction and caramelization has been reduced by ascorbic acid supplementation to a mixture of glucose and ammonia, thus preserving the colorant from AA contamination [15].

Abiotic stress caused by variable substances that leads to oxidative stress of the organism has been associated with cellular ionome destabilization resulting in physiological alterations in a variety of model systems [16,17]. The term ionome determines the collection of all ions in a given biological system, as non-metal and metal elements in nutrition play an irreplaceable role in multiple biological processes. Hence, the ion composition of the cell might represent a reliable tool to study physiological responses of the organism to environmental variations, or nutritional status.

A simple eukaryotic model organism, yeast *Schizosaccharomyces pombe*, was used to investigate the role of AA in cell growth, cell morphology, cell antioxidant capacity, ROS generation, ion balance, and cell cycle progression. *S. pombe* is a rod-shaped, non-pathogenic, widely used, convenient model system that shares many functional similarities of biological processes with higher eukaryotes [18,19]. As the underlying mechanism of AA-induced toxicity is often linked to the overproduction of ROS that triggers oxidative stress, in the presented study we have investigated the possible role of a strong antioxidant, and free radical neutralizer, ascorbic acid (AsA, Vitamin C) [20], to prevent the cytostatic and cytotoxic effect of AA. Although the impact of AA on different model systems has been intensively studied, to our knowledge, its influence on the cell cycle progression and ionome homeostasis in *S. pombe* has yet not been elucidated. Thus, in our study, we provided a newly exerted approach to stress response studies through the determination of the ionome balance sustainment.

## 2. Results

### 2.1. The time and Dose-Dependent Effect of Ascorbic Acid on the Cell Growth Intensity and the Impact of Acryl Amide Addition

**Figure 1:**
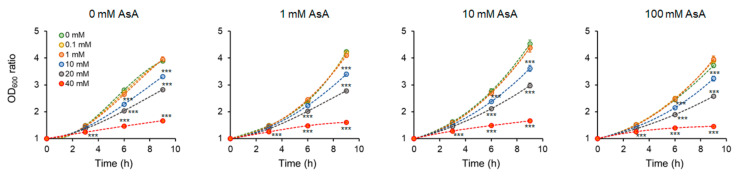
Determination of the cell growth intensity upon AA addition with and w/o AsA pre-treatment. Evaluated was the *OD*_600_ ratio determined every three hours of incubation (3 h, 6 h, and 9 h) and calculated as the difference to the time point 0 h. Cells un- and pre-treated with AsA were subjected to indicated AA concentrations (0 mM green, 0.1 mM yellow, 1 mM orange, 10 mM blue, 20 mM grey, and 40 mM red). Increasing AA concentration led to a marked reduction of the cell growth ability. AsA treatment improved growth ability at 1 mM and 10 mM concentration, while 100 mM did not improve or even reduce the growth of cells. Each point represents the mean value ± SD of 4 individual samples. Statistical differences are indicated as *p* < 0.001 ***.

*S. pombe* cells were grown overnight (o/n) to the exponential growth phase, and *OD*_600_ was adjusted to 0.3. Cells were divided into four groups, one group was left untreated the other three groups were incubated with 1, 10, and 100 mM AsA for 30 min. Afterward, indicated AA concentrations were subjected to the cells and *OD*_600_ was measured every 3 h of incubation. As expected, increasing AA concentrations affected cell growth substantially. Cell treatment with 1 and 10 mM AsA improved the cell growth both, with and without (w/o) AA addition. However, cell treatment with 100 mM AsA either did not affect or reduced the growth of cells, indicating the prooxidative effect of such a high AsA concentration (Figure 1 and Figure S1).

Similarly, the relative cell growth ratio (*RGR*) representing the cell mass gain after every third hour of incubation revealed the positive effect of 1 mM AsA and an even more pronounced positive effect of 10 mM AsA on the cell growth under AA stress, while 100 mM AsA affected the *RGR* negatively (Figure S2). Generation time (gt) that represents the time required for the cell doubling, revealed the positive effect of 10 mM AsA pretreatment, as it shortened the generation time of AA exposed cells, although without reaching statistical significance. Similar to previous observations, 100 mM AsA pre-treatment led to prolongation of the cell growth (Figure S3). For further analyses, the 10 mM AsA concentration was used, as this concentration showed the best protective effect against AA.

To determine the toxic concentration of AA for *S. pombe* cells, the IC_50_ value representing the concentration of AA that leads to 50% cell growth inhibition was calculated (Table 1, Figure S4). Notably, the IC_50_ value is slightly higher under conditions of 10 mM AsA pre-treatment, meaning that a higher AA concentration is required to inhibit the growth of 50% of cells compared to AsA untreated cells, thus confirming the significant role of AsA to protect cells against AA-induced growth alterations.

Determination of cell growth on solid media confirmed the detrimental effect of increasing AA concentration. However, in this particular experiment, AsA pre-incubation was not sufficient to alleviate the negative impact of AA. Cells were allowed to grow on the solid media for 2–3 days, hence as they spent all added AsA they were no longer protected from AA (Figure S5).

### 2.2. Cell Morphology

**Figure 2:**
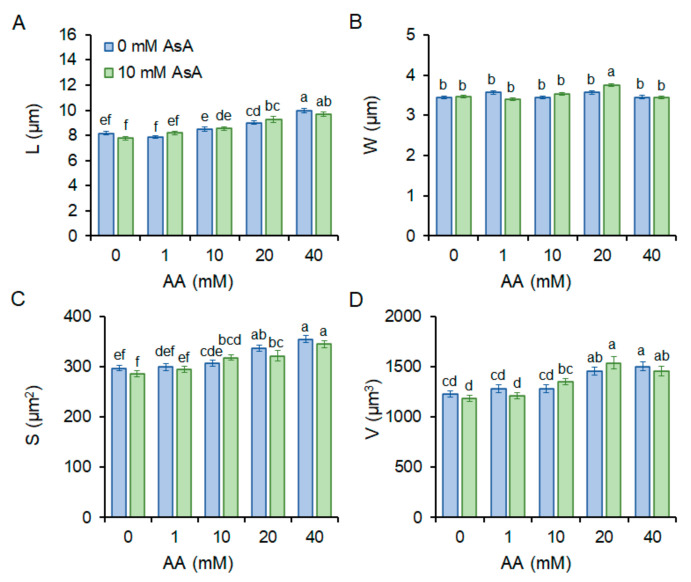
Cell morphology. Length (**A**), width (**B**), surface (**C**), and volume (**D**) were determined in *S. pombe* cells upon addition of indicated AA concentrations, with (green bars) and without (blue bars) AsA pre-treatment. Different letters above bars indicate statistical significance of four independent experiments. Each bar with tick represents mean value ± standard deviation (SD) (*n* = 100). Statistical significance is determined by Duncan’s post-hoc test, different letters above bars indicate statistical difference at a 0.05 level of significance.

As the shape of the single-celled living organism reflects its health condition, we determined changes in the length (µm), width (µm), volume (µm^3^), and surface (µm^2^) of *S. pombe* cells subjected to AA with and w/o AsA pre-treatment. Increasing AA concentration led to cell prolongation, while cell width remained almost constant. Accordingly, cell volume and surface increased with rising AA concentration. Interestingly, pre-treatment of AsA did not significantly improve cell shape compared to untreated cells (Figure 2).

### 2.3. Cell Viability and Metabolic Activity

**Figure 3:**
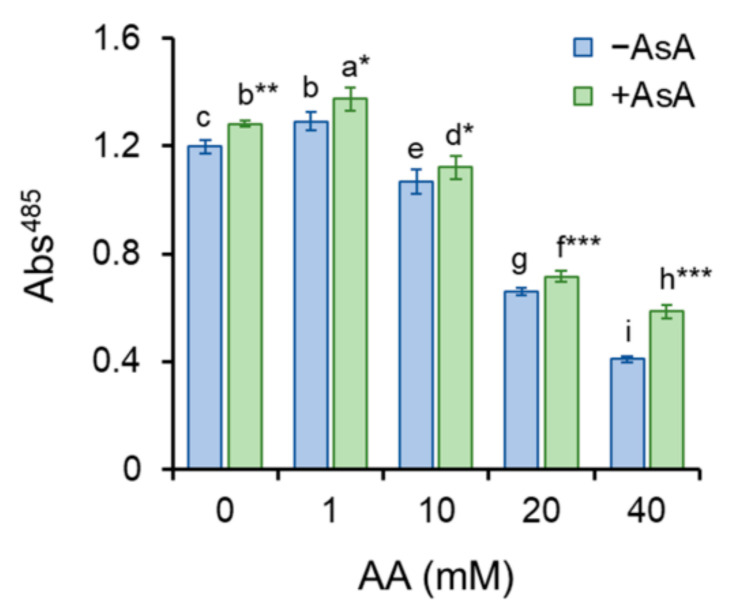
Metabolic activity. Metabolic activity determined as formazan production, detectable at 485 nm and normalized to protein unit, from yellowish tetrazolium chloride (TTC), indicates functionality of mitochondria, thereby indirectly representing cell vitality. Individual bars represent mean value of 8 samples from two independent experiments ± SD. Statistical significance, determined by Duncan’s post-hoc test, was set up as *p* < 0.05 *, 0.01 **, 0.001 *** different letters above bars indicate statistical difference.

Metabolic activity serves as an indicator of mitochondrial functionality. Mitochondria of viable and metabolically active cells reduce TTC to formazan detectable at 485 nm. Enhancement of formazan generation upon stimuli caused by a low concentration of AA (1 mM) indicates excitement of cellular metabolism aiming to protect internal homeostasis. However, higher AA concentrations markedly reduced metabolic activity, suggesting AA-induced deterioration of mitochondrial metabolism and function. Cell pre-treatment with AsA significantly protected mitochondrial function resulting in improvement of metabolic activity (Figure 3). Methylene blue staining is a very useful method to distinguish between viable and dead cells. As it permeates only dead cells, blue-stained cells are considered dead. The percentual proportion of living to dead cells is calculated. Strikingly, although the addition of AA markedly hinders cell growth, cells persist alive trying to survive and overcome the negative effect of AA. Even the highest AA concentration (40 mM) leads to only approximately 4–5% cell death despite almost complete growth retraction. AsA pre-treatment did not cause any significant differences (Figure S6).

### 2.4. Ascorbic Acid Mitigates Aa-Induced Enhancement of ROS Production and Oxidative Stress

**Figure 4:**
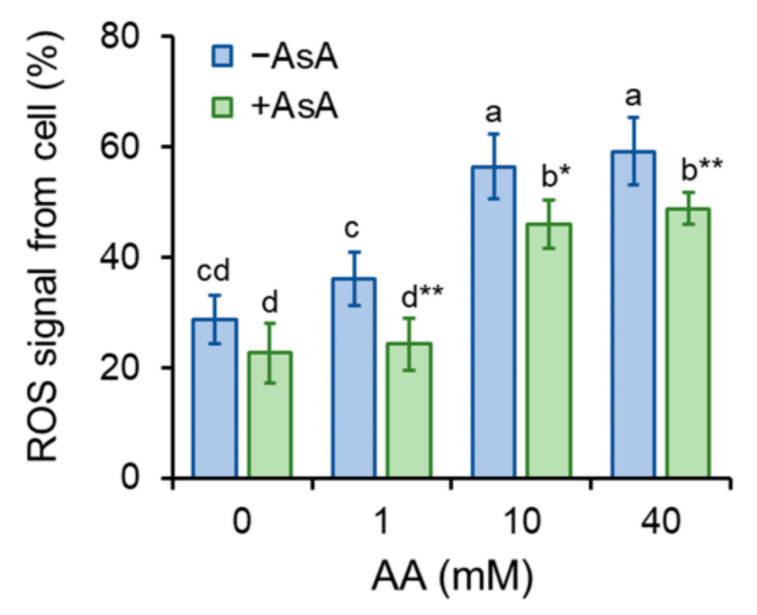
ROS generation. AA-induced enhancement of ROS formation detected as enhancement of fluorescence signal was compared to the control group w/o AA addition and to the group of cells pre-treated with AsA. Individual bars represent mean ± SD of 8 individual samples and two independent experiments. Statistical significance is determined by Duncan’s post-hoc test, different letters above bars indicate statistical difference as *p* < 0.05 *, 0.01 **.

**Figure 5:**
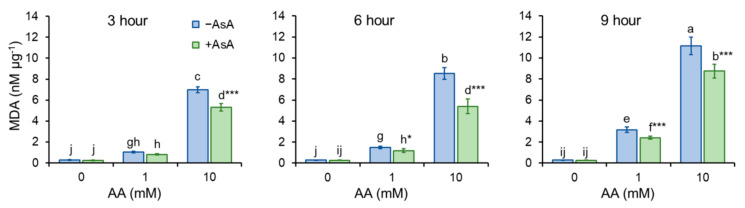
MDA formation. AA triggers oxidative stress resulting in lipid peroxidation. As indicated by enhanced MDA production, AA causes peroxidation of membrane lipids in a dose dependent manner. AsA pre-treatment protect cells from AA-triggered oxidative stress, as it significantly reduces MDA production. Bars represent mean ± SD of 4 individual samples. Statistical significance is determined by Duncan’s post-hoc test, different letters above bars indicate statistical difference as *p* < 0.05 *, 0.001 ***.

Generation of ROS was determined by the use of fluorescence spectroscopy. H_2_DCFDA converts to fluorescent DCF in the presence of ROS, thus serving as an undirect ROS indicator. The addition of AA led to an increase in ROS production. Cell pre-treatment with AsA (10 mM) reduced AA-derived production of ROS significantly (Figure 4). Enhanced ROS formation may lead to oxidative stress. To test this, the formation of malondialdehyde (MDA), the end product of lipid peroxidation, was investigated. As the MDA content is calculated in relation to the protein content, the amount of the protein from the whole-cell extract was determined (Figure S7A). Interestingly, *OD*_600_ values and protein content (µg mL^−1^) showed a positive mutual correlation upon AA and AsA treatment of cells, meaning that the increase in the protein content is related to the cell amount (Figure S7B). As assumed, MDA formation increased upon AA addition, in particular, the addition of 10 mM AA enhanced the generation of MDA dramatically. Cell pre-treatment with AsA reduced MDA formation significantly, thereby protecting cells from AA-mediated oxidative stress (Figure 5).

### 2.5. Acrylamide-Reduced Antioxidant Cell Capacity Is Significantly Restored by AsA

**Figure 6:**
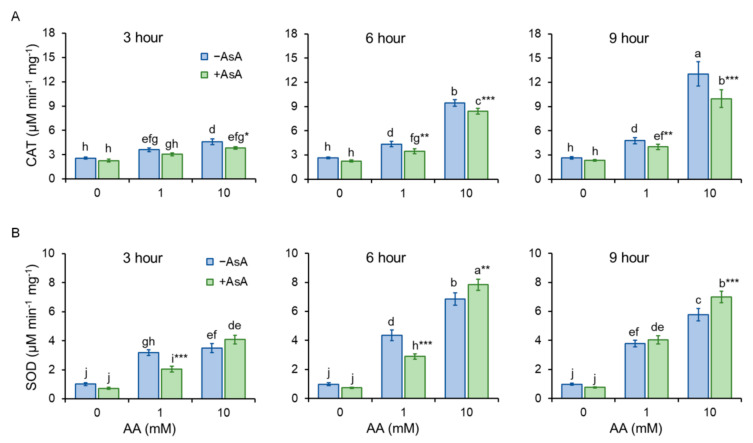
Activity of the antioxidant enzyme system catalase (CAT) and superoxide dismutase (SOD). The antioxidant capacity of cells was determined via detection of antioxidant enzymes activity CAT (**A**) and SOD (**B**). AsA pre-treatment significantly improved AA-altered antioxidant capacity of cells. Bars represent mean ± SD of 4 individual samples. Statistical significance is determined by Duncan’s post-hoc test, different letters above bars indicate statistical difference as *p* < 0.05 *, 0.01 **, 0.001 ***.

Cells undergoing oxidative stress activate protective machineries to eliminate the detrimental consequences of stress. Catalase (CAT) is the enzyme that catalyzes the decomposition of hydrogen peroxide to water and molecular oxygen. Presence of AA in the growth media increases CAT activity in the time and dose dependent manner. AsA pre-treatment protects cells from the negative impact of AA thus CAT activity is significantly lower, compared to the untreated control (Figure 6A). It is worth to mention that CAT activity affects MDA content logarithmically, as the increasing CAT activity reduces MDA content, with the more pronounced positive effect in the samples supplemented with AsA (Figure S8).

Superoxide dismutase (SOD) is the enzymatic antioxidant that helps to maintain redox balance by converting the highly reactive superoxide anion to oxygen and less reactive hydrogen peroxide. Acrylamide at lower concentration (1 mM) induces SOD activation that protects cells from oxidative stress, however, at the same AA concentration, AsA treatment serves as protective antioxidant and defends cells with the need of significantly less SOD activation. Cell incubation with high AA concentration (10 mM) increased SOD activation substantially, AsA pretreatment contributed to the cell protection by additional enhancement of SOD activation (Figure 6B).

### 2.6. Acrylamide Toxicity Triggers Mild Apoptotic Events in S. pombe

As yeast cells exposed to AA displayed an intracellular overload of ROS which is often connected to cell death by apoptosis, double staining with Annexin V-FITC and PI was performed. Annexin V-FITC detects the exposure of PS at the outer layer of the plasma membrane, an early sign of apoptosis. The uptake of PI requires detrimental alteration of the cell membrane referring to its irreversible damage with permanent loss of barrier function, resulting in cell death. Hence, cells that were Annexin V positive and PI negative are considered apoptotic, and PI-positive cells are considered necrotic.

**Figure 7:**
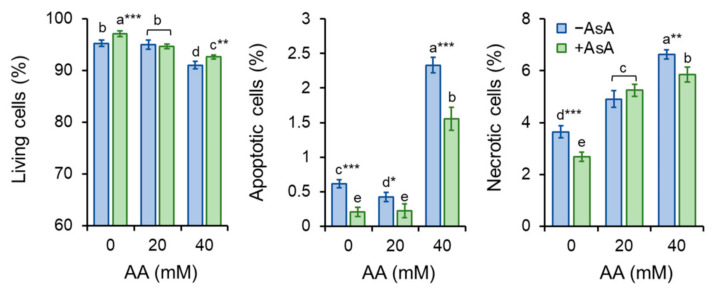
Yeast cell apoptosis determination. To evaluate cell death by apoptosis, Annexin V-FITC signal was determined upon AA exposure of cells un- and pre-treated with AsA. Cells were co-stained with PI to distinguish between apoptosis and necrosis. PI positive cells were considered necrotic. Bars represent mean ± SD of at least 150 counted cells. Statistical significance is determined by Duncan’s post-hoc test, different letters above bars indicate statistical difference. Significance of statistical differences are indicated as *p* < 0.05 *, 0.01 **, 0.001 ***.

Although AA exposure to mammalian cells has been associated with events resulting in programmed cell death, *S. pombe* cells showed only mild signs of apoptosis from AA exposure as only the highest AA concentration (40 mM) enhanced cell apoptosis (Figure 7). Additionally, PI staining revealed only a moderate increase in necrotic cells compared to the untreated control (Figure 7) which is consistent with the cell viability determination by methylene blue staining (Figure S6). Despite the compelling endurance of *S. pombe* cells against AA-triggered death, AsA pre-treatment enhanced cell ability to survive, thus, preserving their vitality.

Altogether, the results show that the loss of cultivability particularly in the presence of lower AA concentration is not connected to cell death as the cell undergoes apoptosis to a similar extent as the control group unexposed to AA.

### 2.7. Acrylamide Triggers Errors in Chromosome Segregation during Cell Cycle

**Figure 8:**
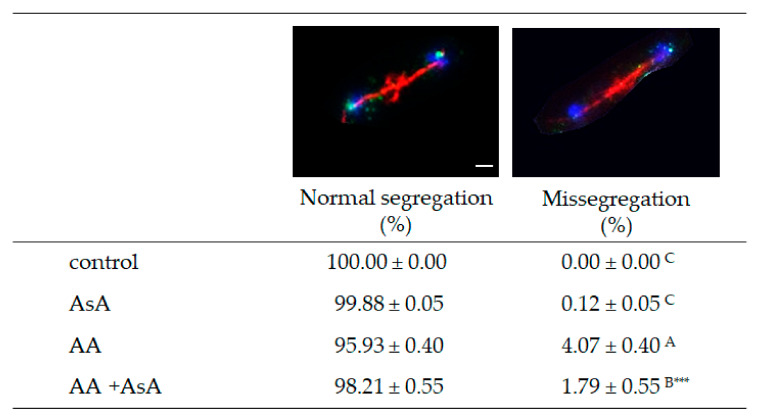
Chromosome segregation during mitosis. Segregation of chromosome II during mitosis was analyzed in cells exposed to 20 mM AA for 6 h with and without 10 mM AsA pre-treatment. Control cells were grown in YES medium without AA and AsA addition. At least 200 cells from 4 inde-pendent experiments were scored for normal or missegregated chromosome II and expressed in percent. Statistical significance is determined by Duncan’s post-hoc test, different letters above bars indicate statistical difference. Significance of statistical differences are indicated as *p* < 0.001 ***.

The cell cycle ensures the growth and development of living organisms, and its error-free progression is thus fundamental for normal life. Segregation of chromosomes during mitosis results in the production of two identical daughter cells from one mother cell. *S. pombe* strain JG 15,457 carries chromosome II marked with GFP (GFP-tagged LacI molecules bind to lacO repeats inserted within the centromere II, cen2-GFP). Segregation of sister chromatids of chromosome II was determined during anaphase in PFA fixed cells 6 h after AA addition. Sister chromatids of the control and AsA pre-treated cells segregated to opposite poles, while AA addition led to sister chromatid non-disjunction in 4% of anaphase cells. However, AsA pre-treatment significantly reduced the non-disjunction of sister chromatids to 1.79% in AA incubated cells. This suggests that AA mediates alterations in the regulation of chromosome segregation most likely through enhancement of ROS production, therefore AsA pre-treatment and its antioxidant property is able, at least to some extent, to protect cells from errors in chromosome segregation (Figure 8).

### 2.8. Ionome Balance

**Figure 9:**
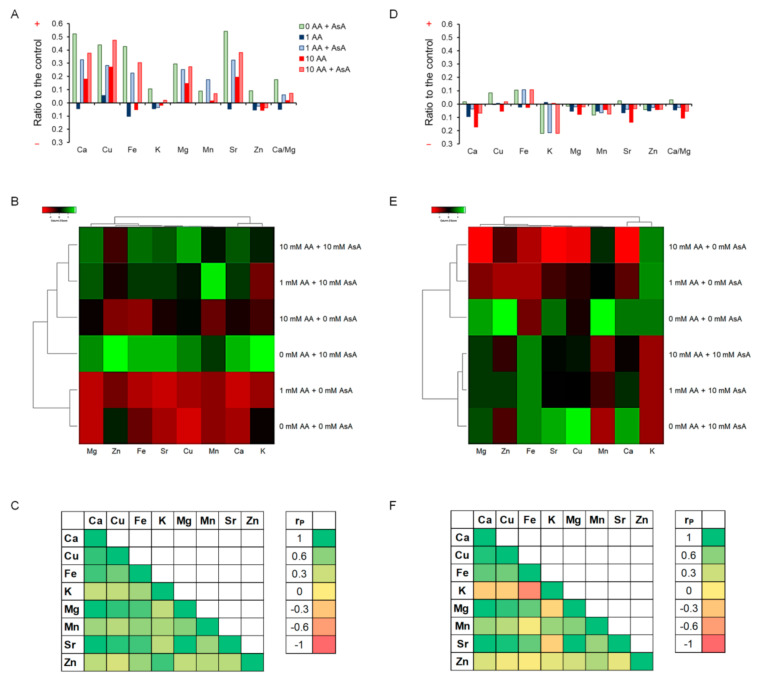
AA mediates disruption of the ionome balance. Differences of the ion content upon treatment are compared to the untreated control after 3 h (**A**) and 9 h (**D**) of incubation and expressed as increase (+) or decrease (−) relative to the control. Control is set up as 0.0 on the x axis. *Z-score* clusters of variables to adjust for the differences in magnitude between different ions after 3 h (**B**) and 9 h (**E**) of incubation. Correlation analyses of mutual ion interactions by Pearson test (r_P_) after 3 h (**C**) and 9 h (**F**) of incubation.

Maintaining intracellular ionome homeostasis is an important mechanism by which cells adapt to the stress caused by cell disrupting agents such as AA. Accordingly, AA-mediated cell homeostasis alterations triggered an imbalance in the intracellular mineral element content. Relative to the untreated control, 1 mM AA exposure for 3 h caused a decrease in the content of most of the evaluated elements, except for Cu, while cells exposed to 10 mM AA displayed increased (Ca, Cu, Mg, Mn, Sr) or slightly decreased (Fe, K, Zn) ion concentrations. Marked enhancement of all determined element levels was detected upon cell pre-incubation with AsA as compared to the untreated control. This, in turn, resulted in the increase in the ion content of cells co-treated with AA and AsA compared to AA-exposed cells revealing the positive impact of AsA treatment in the protection of ion homeostasis maintenance (Figure 9A). The second messenger Ca was the dominant component that accumulated in the cell to prevent the ionome balance disruption. Strikingly, Sr content increased in the same fashion as the Ca level possibly due to utilization of the open Ca channels. Changes in levels of ions related to the enzymatic antioxidant system (Cu, Mn, Zn) might be connected to AA-triggered ROS overproduction and oxidative stress. Variables standardization by *Z-scores* reveals that in general, AsA treatment led to enhancement of the concentration of most of the analyzed elements compared to AsA untreated cells (Figure 9B). Moreover, correlation analyses show positive mutual interactions among all tested elements after 3 h of AA exposure (Figure 9C). A different situation was observed after 9 h of incubation, as most of the evaluated mineral elements showed reduced concentration relative to the untreated control (Figure 9D). The most prominent drop in the concentration was observed with K of AsA preincubated cells, which is probably related to the form of ascorbate used in our study, the Na salt. After 9 h of incubation, all AsA was spent, therefore K was no longer required to compensate for the boost of Na. Interestingly, AsA treatment led to enhancement of Fe levels resulting in Cu regulation. Standardized *Z-scores* clearly created clusters of cell responses dependent on the treatment revealing that the treatment is the variable responsible for changes in the ion content (Figure 9E). Ion interactions display positive mutual correlations, except for K due to its marked drop in concentration. The most prominent positive correlation of all tested ions emerged toward Ca (Figure 9F). Altogether our results clearly show that AA exposure causes marked ion balance disruption in the cell that leads to cell homeostasis alterations. However, to our surprise, yeast cells subjected to long-term AA exposure show signs of adaptation to the toxicant.

## 3. Discussion

Acrylamide is a chemical substance often used in industry, however, with a potential unintended human exposure. Due to the reported risks linked with its use, the effect of its exposure to eukaryotic cells requires adequate assessment. In the present study, we used fission yeast *Schizosaccharomyces pombe* as a eukaryotic model organism to investigate the effect of AA-induced oxidative stress on chromosome segregation during mitosis. Yeast *S. pombe* represents a convenient model system for elucidating a variety of biological processes including growth, division, metabolic activity, and antioxidant capacity of the organism. This nonpathogenic single-celled eukaryote is easy to grow and manipulate. Additionally, its genome contains protein-coding genes responsible for cell division and cellular organization, which are also found in the genome of higher eukaryotes. Hence, it became a popular model system to study the basic principles of a cell that can be used to understand more complex organisms [21,22]. The eukaryotic mitotic cell cycle is a highly complex event that guarantees duplication and faithful segregation of all components required for cell survival to daughter cells. To ensure genomic integrity, the error-free DNA synthesis phase (S-phase), and the mitosis phase (M-phase) are required to occur exactly once per cell cycle [23]. Progression of the cell cycle is regulated by a variety of not only internal but also external factors. Within the last decades, attention has been paid to reactive oxygen species (ROS) that seem to play an indisputable role in the regulation of cell cycle progression [24,25]. Although ROS are important mediators of natural physiological processes, their overproduction contributes to oxidative stress that might cause cell damage through oxidization of physiologically important biomolecules and alteration of cell signaling pathways [26,27]. Despite the relative resistance of *S. pombe* cells toward AA toxicity determined by the IC_50_ value (Table 1), the addition of AA to growth media resulted in the dose and time-dependent growth retardation (Figure 1) and AA-mediated enhancement of ROS production (Figure 4). This is related to the cellular metabolic activity, which was at first slightly stimulated upon 1 mM AA concentration, whereas higher concentrations of AA dramatically reduced the mitochondrial functionality (Figure 3). Similarly, many studies on a variety of model systems showed that AA decreases cell viability, induces protein modifications, increases the production of ROS, and alters mitochondrial function [26,28,29,30]. Therefore, to counteract the toxic impact of AA, we investigated the possible protective effect of a strong natural antioxidant, ascorbic acid (AsA, Vitamin C). Growth of AA exposed cells upon pre-treatment of AsA significantly improved, however only 1 mM and even more pronounced 10 mM concentrations were able to improve cell growth, 100 mM AsA caused growth alterations, suggesting pro-oxidative properties of such high concentration. Hence, 10 mM AsA was used throughout the experiments. The significant positive effect of AsA pre-treatment resulted in the reduction of ROS production, and improved metabolic activity. Other authors tested various compounds with antioxidant properties to eliminate AA toxicity. Jackfruit flake digest and a naturally occurring flavonoid myricitrin reduced excessive ROS production resulting in alleviation of AA-triggered mitochondrial disorders in Caco-2 cells [31,32], in HepG2 cells AA-induced ROS overproduction was reduced by curcumin or mulberry digest [33,34]. As ROS-induced oxidative stress often triggers membrane lipid peroxidation, we determined the level of MDA, the end product of lipid peroxidation, upon AA addition. MDA content increased significantly even after cell exposure to 1 mM AA, while 10 mM AA led to marked enhancement of MDA levels. Notably, cell pre-incubation with AsA for 30 min led to significant protection against oxidative stress resulting in the reduction of MDA content. These results are supported by a previous study showing that AsA reduces enhanced ROS production or MDA content triggered by the AA metabolite, glycidamide, and exposure to Sertoli cells [35]. The enzymatic antioxidant defense system of the cell is activated upon exposure of the organism to oxidative stress. The activity of the superoxide scavenging enzyme SOD which converts superoxide radicals to less toxic H_2_O_2_ and CAT which catalyzes the direct decomposition of H_2_O_2_ to H_2_O was determined every three hours after cells were exposed to AA. Our results reveal that 1 mM AA enhances the formation of superoxide already after 3 h of exposure, as the SOD activity increases significantly. Interestingly, AsA pre-treatment reduced the formation of superoxide, as the activity of SOD is lower as compared to AsA untreated cells. Cell treatment with 10 mM AA for 3 h led to increased SOD activity of both pre-treated and untreated cells with AsA suggesting the supportive role of AsA in cell protection against AA toxicity. Six hours of cell incubation with AA led to a noticeable enhancement of SOD activity with AsA protective effect similar to that observed at three hours of incubation. Apparently, after 9 h of AA exposure, the formed superoxide was neutralized as the SOD activity decreased as compared to its activity after 6 h of incubation (Figure 6B). In accordance with our observations, the SOD activity of human erythrocytes exposed to AA increased in a dose-dependent manner [36]. We assume that due to SOD-triggered superoxide radical decomposition to H_2_O_2_, CAT activity increased upon AA exposure. Its activity depended on the time of exposure and the dose of AA. AsA pre-treatment reduced the CAT activity of AA exposed cells, as its own antioxidant activity protected cells, and the CAT activity was therefore not required at such a high level (Figure 6A). Similarly, Celik et al. [37] detected increased CAT activity upon AA addition to HEK293 cells, although the activity of the enzyme was dependent on the dose of AA exposed to cells. Several scientific publications present decreased SOD and CAT activity of various model systems after AA exposure. This might result from the different behavior and sensitivity of the used model organism toward AA, different doses, and exposition times of AA, and/or due to manipulation of the model organism that already caused stress. However, the remaining common characteristic is the protection of the model organism against AA-induced oxidative stress via substances with antioxidant properties [38,39,40]. Taken together, the presented results showed that AsA pre-treatment significantly reduces ROS levels and promotes SOD and CAT activity to protect cells exposed to AA. Rod-shaped *S. pombe* cells are normally 6–7 µm in length and 2–3 µm in width. Before undergoing the G2–M transition, they grow until their length reaches approximately 14 µm. However, the size at which cells enter mitosis largely depends on environmental stimuli such as nutrient availability or stress. Poor nutrient conditions force cells to divide at a smaller size than in nutrient-rich media. Cell exposure to challenging conditions initiates signaling through the regulatory network, so the shape of cells is often the result of the prior signaling. The size modulation typically occurs through the MAP (mitogen-activated protein) kinase and TOR (target of rapamycin) signaling pathways acting on Cdc25 [19,41]. AA exposure significantly modulated the shape of *S. pombe* cells in a dose-dependent manner, the higher the concentration, the bigger the cell (Figure 2). Interestingly, AsA pre-treatment had only minor effects on the cell shape regulation of AA-exposed cells.

Strikingly, AA exposure in a dose close to IC_50_ value (40 mM) hindered cell growth substantially, however, the cell’s vital functions and its metabolism were preserved as only approximately 3–4% of cells were unviable (Figure S4). Moreover, *S. pombe* cells show fairly high resistance against AA-triggered cell death by apoptosis (Figure 7). This suggests that despite attenuated cell vitality, most of the cells are able to survive. A negative condition of such survival is the accumulation of cell cycle regulation alterations as AA exposure leads to marked cell cycle delay and errors in chromosome segregation (Figure 8). The entry of mitosis is a strictly regulated process that controls the attachments between kinetochores and microtubules through sophisticated pathways including error correction (EC) and spindle assembly checkpoint (SAC) pathways. The Aurora B kinase, Ark1 in *S. pombe*, is the major kinase of these pathways that facilitates the establishment of accurate kinetochore–microtubule attachments. Alterations of its function have been associated with frequent occurrence of sister chromatids non-disjunction during mitosis [42,43] Hence, we assume that AA might negatively influence Ark1 function that in turn leads to erroneous segregation of chromosomes. However, Sickles et al. [44] also presented a potential mechanism of acrylamide-induced disruption of cell cycle mitotic activity that results in genomic instability. According to their study, the microtubule-depolymerizing kinesins may be inhibited by AA, which results in the failure of the migration of chromosomes from the metaphase plate. Similarly, Adler et al. [45] already in 1993 in their report suggested that AA might induce aneuploidy in mammalian cells through concentration-dependent spindle disturbances resulting in improper functioning of the spindle. Many studies showing AA altered cell cycle [46,47] are consistent with our findings that AA induces improper segregation of chromosomes and thus reduces mitotic activity. However, the underlying mechanism of its impact on cell cycle regulation remains unclear. One of the most important mechanisms of AA-induced toxicity is oxidative stress caused by ROS overproduction. As AsA pre-treatment reduced chromosome missegregation events we suggest that ROS-derived alterations of the cell cycle regulators are responsible for the alteration of mitotic chromosome segregation.

Ionome is critical for a variety of biological processes including growth or defense responses however, conditions for the regulation of the ionome homeostasis in the fission yeast under stress are only partially known. Despite the conserved role of each particular element, the yeast ion composition varies according to changes in the surrounding environment. As we have shown previously, acute contamination by Cd or Ni causes not only dramatic changes in the ion content of the cell but also affects ion trafficking through mutual mineral element interactions [48]. In line with this, AA-triggered stress results in ion balance disruption in *S. pombe* cells, leading to changes in mineral element concentrations of cells exposed to AA (Figure 9A,D). Cell response to AA toxicity resulted in a reduction in Fe levels which leads to an increase in Cu content. In addition, levels of Cu, Mn, and Zn ions that are involved in the antioxidant defense system of the cell were modulated by AA exposure, hence supporting AA-induced oxidative stress. Moreover, AA exposure caused accumulation of the second messenger and enzyme co-factor Ca, in order to reduce the negative impact of AA on the cell. Similarly, an influx of Ca from extracellular sources was reported by Popa et al. [49] in *Saccharomyces cerevisiae* cells exposed to high levels of oxidative stress. The authors concluded that Ca overload upon oxidative stress mediates the cytotoxic effect of the stressor rather than serving as an adaptation mediator for the cell. However, we believe that Ca protects cell viability as cells exposed to AA concentrations that almost completely hinder cell growth; only a small percent of such cells underwent apoptosis or lost vitality. In accordance, cell co-treatment with the strong antioxidant, AsA, caused a marked boost in Ca levels. This implies that Ca uptake is involved in the reduction of oxidative stress, thus protecting cells from AA toxicity. In line with this, in our previous study, we described the positive effect of AsA against another toxicant causing oxidative stress that disrupts the ionome, cadmium (Cd) [50]. AA-induced perturbations in the ionome led to changes in mineral elements’ content and their mutual interactions in the dose in a time-dependent manner. Long-term exposure to AA led to downregulation of previously upregulated ion contents indicating initialization of cell adaptation (Figure 9A–F). Cell homeostasis, however, largely depends on the ionome balance referring to the complexity of the regulation of its sustainability. Over 600 genes in the yeast genome have been described to significantly impact the ionome. Clustering based on the ionome phenotype identified genes that target physical and genetic interaction networks between genes within a particular mineral nutrient regulatory function [51]. Thus, a possible explanation of the AA’s ability to alter ion balance might arise from the AA-mediated changes in the expression of transporter genes resulting in ion accumulation in the cell although the exact mechanism still needs to be elucidated.

## 4. Materials and Methods

### 4.1. Yeast Cultivation and Growth Conditions

The wild-type *Schizosaccharomyces* *pombe* strain *SP72 h+ ade6-M210 ura4-D18 leu 1–32* was used for all studies except for the immunostaining experiment, for which the *JG 15,457* strain (cen2(D107)::KanR-ura4 + -lacO his7 + ::lacI-GFP) carrying chromosome II labeled with GFP was used. Yeast cells were cultured in the complete YES liquid medium containing 0.5% yeast extract, and 3% glucose, supplemented with 225 mg L^−1^ of amino acids (adenine, L-histidine, L-leucine, L-lysine, and uracil) at 30 °C, under aerobic conditions with vigorous shaking (150 rpm). The fission yeast grows at temperatures ranging from 18 to 37 °C with the optimum at 30 °C [52]. To avoid the collateral effect of the restrictive growth conditions and the AA-mediated effect on the cell behavior, cells were cultured at the optimum temperature and on the complete nutrient media.

### 4.2. Growth Intensity Determination

Cells from the overnight (o/n) culture were divided into four equal portions, three of which were supplemented with either 1, 10, or 100 mM of L-ascorbic acid (AsA) (Sigma–Aldrich, St. Louis, MO, USA), respectively, diluted in distilled water, for 30 min, while the third was left untreated, and incubated at 30 °C and 150 rpm. The density of cultured cells was then adjusted to an *OD*_600_ = 0.3, transferred to 24 well plates, and treated with various concentrations of AA (0, 1, 10, 20, 30, and 40 mM). Optical density at 600 nm of the culture incubated at 30 °C and 150 rpm for 9 h was measured every 3 h by Glomax Multi Detection System (Promega Corporation, Madison, WI, USA). Hence, as one life cycle of *S. pombe* takes approximately 3 h, the cell growth intensity upon AA addition was analyzed within three life cycles. The total growth intensity (*OD*_600_ ratio) is calculated as a ratio in the cell density at 3 h, 6 h, or 9 h, respectively, to the 0 h time point [41].

### 4.3. Relative Growth Rate (RGR) and Generation Time (gt)

*RGR* represents the cell mass gain determined as the increase in the *OD*_600_ value after each third hour of incubation and is calculated by Equation (1):

(1) $$RGR=\frac{log\left(\Delta OD_{600}\right)}{\Delta t}$$

where Δ*OD*_600_ represents the difference between optical cell density measured at time *t* + 1 and previous time *t*, and Δ*t* is the time interval (3 h).

Generation time (*gt*) represents cell doubling time and is calculated by Equation (2):

(2) $$gt=\frac{log\;\left(2\right)}{m}$$

where *m* is the gradient of the regression line.

### 4.4. IC_50_ Value

Represents concentration of AA that leads to 50% cell growth reduction. To calculate the IC_50_ value, cells were treated with serially diluted AA using log 2 dilution starting at 400 mM. Cells without (w/o) AA treatment were used as a control. Cells were incubated in 96 well plates for 9 h at 30 °C w/o shaking, and *OD*_600_ was determined every hour. For the calculation, the freely available online calculator was used, Quest Graph™ IC50 Calculator (https://www.aatbio.com/tools/ic50-calculator/, last accessed on 22 November 2021; AAT Bioquest, Sunnyvale, CA, USA).

### 4.5. Characterization of the Yeast Morphology

Yeast morphology analysis was performed as previously described [50]. Briefly, cells incubated with AA for 9 h with and w/o ASA pre-treatment were visualized under bright-field microscopy at 40× magnification (Leica DMI 6000, Leica microsystems, Wetzlar, Germany). Microscopy images were captured, and cell morphometric analyses were determined by the ImageJ software v. 1.52r (National Institutes of Health, CA, USA).

Cell volume (*V*; µm^3^) was calculated according to Equation (3):

(3) $$V=\frac{4}{3}\pi LW^{2}$$

where *L* represents the cell length, and *W* is the cell width.

Cell surface (*S*; µm^2^) was calculated according to Equation (4):

(4) $$S=2\pi \left(W^{2}+LW\frac{arcsin\varepsilon }{\varepsilon }\right)$$

where, factor *ε* is calculated as $\varepsilon =\frac{\sqrt{L^{2}-W^{2}}}{L}$.

### 4.6. Spot Test

To solid YES media 0, 0.1, 1, 10, and 20 mM of AA was added. Cells from the overnight culture were divided into two groups, one was left untreated, and the second was pre-treated with 10 mM AsA. Serially diluted cells resulting approximately in 10,000, 1000, 100, and 10 cells/spot, were placed on plates. After 2–3 days of incubation at 30 °C, the size and density of spots were compared.

### 4.7. Preparation of the Cell Extract for Biochemical Analyses

Control and AA-treated cells with and w/o AsA preincubation were collected by centrifugation at 8500 rpm for 90 s, washed 3 times with sterile H_2_O, and resuspended in PBS (pH 7.0). Cells were either directly used for further analyses or stored at −80 °C. Cell homogenization was achieved by sonication (Digital Sonifier 450, Branson Ultrasonics Corp, Danbury, CT, USA) at 3 × 30 s intervals (repeated 1s pulses followed by a 1 s pause giving 15 pulses within 30 s, and power 80 W representing 20% of the full power capacity) on ice. Cell debris was removed after 15 min centrifugation at 14,000× *g* and 4 °C. In the collected supernatant the protein level, metabolic activity, catalase (CAT) activity, superoxide dismutase (SOD) activity, and malondialdehyde (MDA) content were determined.

### 4.8. Metabolic Activity

Metabolic activity of yeast was calculated according to [53] with a few modifications. Briefly, yeast suspensions were centrifugated for 90 s at 10,000× *g* and washed with PBS (pH 7.0). Pellets were resuspended in 1 mL 0.5% 2,3,5-triphenyltertrazolium chloride (TTC) diluted in PBS and incubated for 20 h in the dark at 30 °C. After incubation, the pellet was washed twice with PBS, and generated red formazan was extracted by addition of 1 mL ethanol:acetone (2:1) mixture prior to cell lysis by sonication. Absorbance was measured at 485 nm and metabolic activity was calculated as relative units (r.u.) of absorbance per mg protein.

### 4.9. Cell Viability

Cells un- and pre-treated with AsA exposed to AA for 6 h were washed in PBS and 0.05% of methylene blue (Sigma–Aldrich) with 0.1% natrium citrate (Sigma–Aldrich) was added for 5 min. Methylene blue is able to penetrate only dead cells, hence blue-stained cells are considered as dead. Microscopic slides were prepared and a percentual portion of the dead to living cells was determined.

### 4.10. Biochemical Analysis

Agilent Cary 60 UV/VIS spectrophotometer (Agilent Technologies, Santa Clara, CA, USA) was used to determine CAT activity represented as the stepwise decrease in the absorbance at 240 nm for 90 s which determines the H_2_O_2_ decomposition. Addition of 50 µL of 30 mM H_2_O_2_ to 100 µL of the solution containing sample initialized reaction; the final volume of the reaction was 600 µL. The molar absorption coefficient of 36 mM^−1^ cm^−1^ was used to calculate specific catalase activity.

Total superoxide dismutase (SOD) activity was assayed according to [54] with slight modifications. Briefly, 100 µL of homogenized sample solution was added into 880 µL of a reactive mixture of 50 mM phosphate buffer (pH 7.8) containing 1 mM EDTA, 13 mM L-methionine, and 75 µM NBT (nitroblue tetrazolium). Finally, 20 µL of 2 mM riboflavin was added and the reaction was started by light irradiation (5000 lux) for 10 min at 20 °C. Absorbance of the samples was measured spectrophotometrically at 560 nm.

Malondialdehyde (MDA) content that represents lipid peroxidation was evaluated as previously described by [50]. Briefly, the TBA solution (15% trichloroacetate (TCA) containing 0.375% (*w*/*v*) thiobarbituric acid (TBA) was added to the supernatant of each sample and incubated at 95 °C for 30 min. The sample was rapidly cooled on ice and centrifuged at 8500 rpm for 60 s, the absorbance of the supernatant was measured at 532 and 600 nm at the Agilent Cary 60 UV/VIS spectrophotometer. The molar absorption coefficient 153 mM^−1^ cm^−1^ was used to calculate MDA content in nmol µg^−1^ protein.

The Bradford assay [55] was used to determine protein concentration at 600 nm using bovine serum albumin (Sigma–Aldrich, St. Louis, MO, USA) as a standard.

### 4.11. Determination of ROS Generation

Generation of the total ROS was performed as previously described by [56] with slight modifications. Control and AA-treated cell cultures with and w/o AsA (10 mM) preincubation were adjusted to *OD*_600_ = 1, washed with PBS, and incubated with 10 µM H_2_DCFDA (Sigma–Aldrich) at 30 °C in the dark for 1 h without shaking. H_2_DCFDA is a compound that is oxidized by ROS to a highly fluorescent DCF, which is fluorescently detectable at 498 nm wavelength, thus providing an estimate of ROS levels in the cell. The excessive fluorescent dye was washed off and the cells were resuspended in PBS. Fluorescence filter with 490 nm excitation wavelength at the Glomax Multi Detection System (Promega Corporation, Madison, WI, USA) was used for the DCF fluorescence detection. The measured values were normalized to the untreated cells.

### 4.12. Detection of Apoptosis and Necrosis

Evaluation of the yeast cells’ apoptosis was performed according to [57] with some modifications. Annexin V- FITC (fluorescein isothiocyanate) -conjugated that specifically binds to phosphatidylserine (PS) residues, was used to detect the externalization of PS which is an apoptosis marker. Propidium iodide (PI) (Sigma Aldrich) penetrates dead cells and serves as a marker to differentiate between apoptosis and necrosis. After 1 h AA exposure with and without AsA treatment, yeast cells were collected by centrifugation and washed twice with phosphate-buffered saline (PBS), pH 6.8. Washed cells were resuspended in sorbitol buffer (1.2 M sorbitol, 0.5 mM MgCl_2_, and 35 mM K_2_HPO_4_), pH 6.8 to a final concentration of 1 × 10^7^ cells mL^−1^. For cell wall digestion, cells were incubated with 10 µg mL^−1^ zymolyase (Roche) in sorbitol buffer for 1 h at 37 °C. Afterward, 1 mL of spheroplasts were centrifuged at 500 rpm for 5 min and resuspended in 60 µL of incubation buffer (containing the Annexin-V-FITC and PI) and incubated for 10 min at room temperature (RT). Cells were visualized by the fluorescence microscope (Leica DMI 6000, Leica microsystems, Wetzlar, Germany). Two independent experiments were evaluated, each containing at least 150 cells. Cells were observed in the stepwise-selected microscope fields to avoid counting the same cells.

### 4.13. Determination of Ion Composition

Ion composition was evaluated by the use of ICP-OES (ICP-OES 720, Agilent Technologies Australia (M) Pty Ltd., Santa Clara, CA, USA) as previously described [48]. Briefly, yeast cells exposed to 0-, 1-, and 10-mM AA for 3 and 9 h with and without AsA pre-incubation were washed three times with deionized water and incubated at 55 °C for 12 h. Weighted yeast pellets were placed into PTFE digestion tubes and 3 mL of extra pure HNO_3_ was added for pressure microwave digestion by the ETHOS-One (Milestone, Srl., Sorisole (BG), Italy) microwave digestion system. Mineralized samples were filtered through a quantitative Munktell filter paper No. 390 (Munktell & Filtrak, Bärenstein, Germany) into 25 mL volumetric flasks and filled with deionized H_2_O in 4 biological replicates. Afterwards, the ion content detected by ICP-OES was calculated to µg g^−1^ of the yeast dry matter and expressed as the content ratio to the untreated control.

### 4.14. Immunostaining and Fluorescence Microscopy

Chromosome segregation was analyzed by the use of fluorescence microscopy as previously described [58]. Shortly, yeast strain *JG15457* with the chromosome II marked with GFP was grown in YES medium at 30 °C and 150 rpm to achieve exponential growth. Cells were collected after 6 h of incubation, fixed by 2% PFA, and stained with primary TAT1 mouse monoclonal anti-tubulin and rabbit polyclonal anti-GFP antibodies, DNA was visualized using DAPI. Analyses were performed at the fluorescence microscope (Leica DMI 6000, Leica microsystems, Wetzlar, Germany) equipped with a digital camera. At least 200 cells in the anaphase stage of the mitotic cell cycle were evaluated for correct or incorrect segregation of chromosome II.

### 4.15. Statistical Analysis

Data are expressed as the mean ± standard deviation (SD). Statistical significance of obtained differences was analyzed by the ANOVA Duncan’s and Fisher LSD post-hoc test using the Statistica 10 software (StatSoft Inc., Tulsa, OK, USA). Lavene’s and Cochran’s tests were used to evaluate data homogeneity and normality distribution of the results. Limits of the statistical significance were set up to *p* < 0.05 *, 0.01 **, 0.001 ***. Time-dependent ionome changes were calculated for each ion as a ratio to the untreated control. Absolute ion concentrations were then *Z-score*-transformed to adjust for the differences in magnitude between different ions according to Formula (5):

(5) $$Z-score^{Individual}=\frac{\left(Concentration^{individual}-Mean\;concentration\right)}{SD}$$

*Z-scores* were used for cluster analysis by the average linkage clustering method with Pearson distance measurement. Pearson correlations between individual ions were calculated. *Z-score* and ion correlations were visualized by Heatmapper [59].

## 5. Conclusions

In this study, we investigated the underlining toxic effect of AA on cell vital functions by multiapproach analyses covering large-scale biological processes and stress responses. AA-induced enhancement of ROS production led to oxidative stress in *S. pombe* resulting in cell cycle arrest and alterations in chromosome segregation. Additionally, the cell disturbing activity of AA was confirmed by its negative effect on ion balance maintenance. Supplementation of ascorbic acid significantly protected cells against AA-mediated cytotoxicity due to its direct ROS scavenging activity and intracellular Ca uptake support. Our complex study extended the knowledge of AA-induced toxicity from the known metabolic disorders to disrupted ionome balance and altered chromosome segregation resulting in cell cycle arrest without marked signs of apoptosis. Additionally, we show that AsA treatment not only reduced ROS production but also improved ionome homeostasis and prevented errors in chromosome segregation during mitosis. Though, our results suggest that the use of AsA, a natural antioxidant supplementation, could significantly attenuate AA-induced cytotoxicity which might have health-protective implications in situations of dietary AA exposure. However, as AA is known to accumulate in the cell, further investigations of low-dose AA exposure for a longer period are required.

## Figures and Tables

**Table 1:** IC_50_ value indicating inhibitory concentration of AA to cell growth.

AsA	IC_50_ (mM of AA)	R^2^
0 mM	30.8337	0.932
10 mM	37.8238	0.916

R^2^—coefficient of determination.

## Data Availability

The authors confirm that the data supporting the findings of this study are available within the article and its Supplementary Materials.

## Supplementary Materials

The following are available online at https://www.mdpi.com/article/10.3390/molecules27134307/s1, Figure S1: Cell growth intensity upon AA addition with and w/o AsA pre-treatment, Figure S2: Relative growth rate of cells ex-posed to AA with and w/o AsA pre-treatment, Figure S3: Generation time (gt), Figure S4: Determination of IC50 upon AA exposure with and w/o AsA pre-treatment, Figure S5: Spot test, Figure S6: Cell viability upon AA addition with and w/o AsA pre-treatment, Figure S7: Protein content, Figure S8: CAT/MDA ratio.

## Sample Availability

Samples of the compounds are available from the authors or from commercial sources.
