# Supplementary material for: Acrylamide-Derived Ionome, Metabolic, and Cell Cycle Alterations Are Alleviated by Ascorbic Acid in the Fission Yeast
Source: Molecules. 2022 Jul 5;27(13):4307. doi: 10.3390/molecules27134307 (PMC9268660; doi:10.3390/molecules27134307)
Supplement: Supplementary file 1 [file molecules-27-04307-s001.zip › molecules-1797551-supplementary.pdf]

Supplementary material

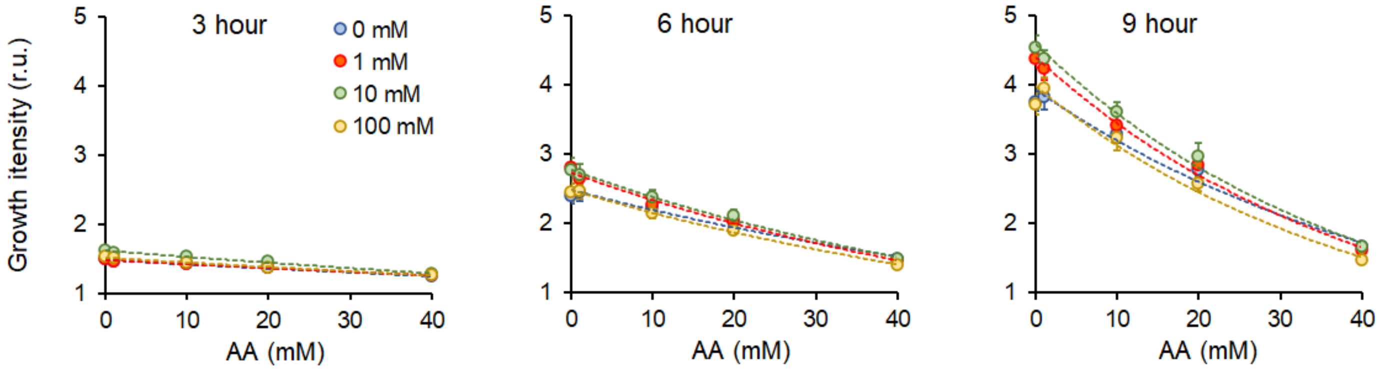

Figure S1. Cell growth intensity upon AA addition with and w/o AsA pre-treatment. Evaluated was the OD_{600} ratio determined every three hours of incubation (3 h, 6 h, and 9 h) and calculated as the difference to the time point 0 h. Cells pre-treated with AsA (0 mM blue, 1 mM red, 10 mM green, and 100 mM yellow) were subjected to indicated AA concentrations. Increasing AA concentration led to marked reduction of the cell growth ability. AsA treatment improved growth ability at 1 mM and 10 mM concentration, while 100 mM did not improve or even reduced growth of cells. Each point represents mean value ± SD of 4 individual samples.

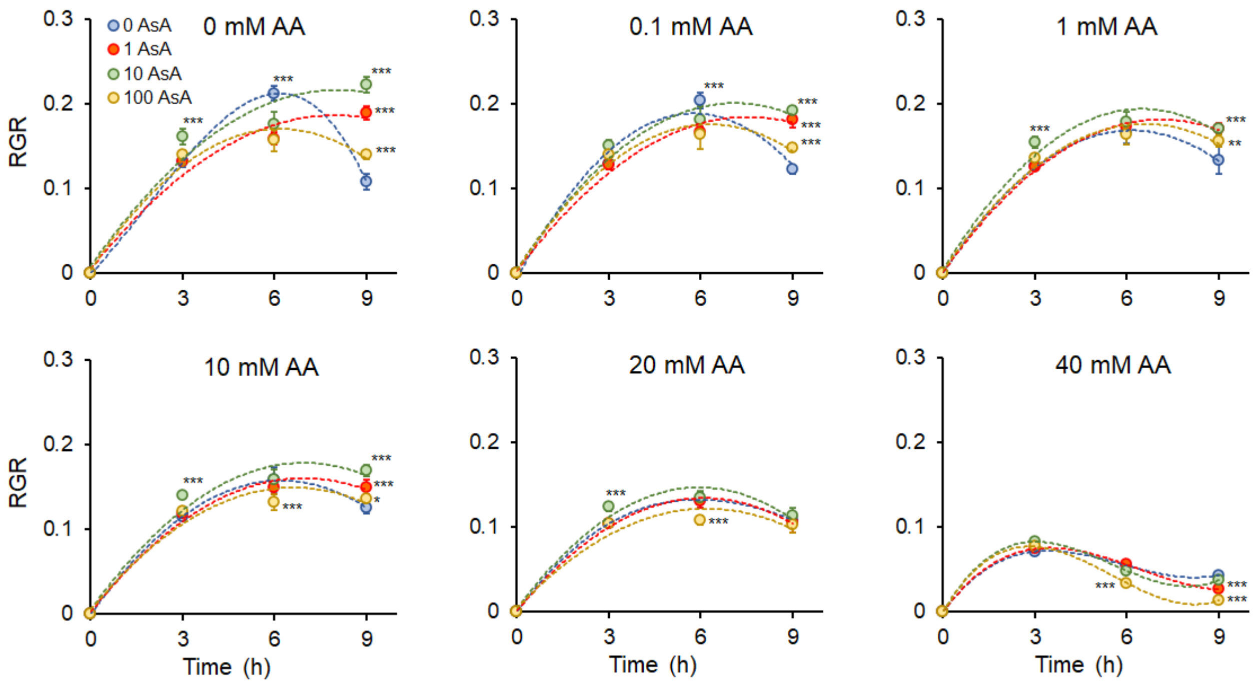

Figure S2. Relative growth rate (RGR) of cells exposed to AA with and w/o AsA pre-treatment. Evaluated was the OD_{600} ratio determined every third hour within 9 h of incubation. Detected cell mass gain confirmed the positive effect of 1 and even more pronounced positive effect of 10 mM AsA on the cell growth under AA stress. RGR was negatively affected by 100 mM AsA treatment. Each point represents mean value ± SD of 4 individual samples. Statistical differences are indicated as $p < 0.05$ *, 0.01 **, 0.001 ***.

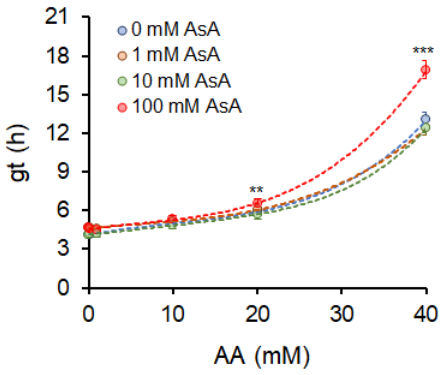

Figure S3. Generation time (gt), the time required for cell doubling. AA addition substantially increased time required for cell doubling in a dose dependent manner. AsA pre-treatment (0 mM blue, 1 mM orange, 10 mM green, and 100 mM red) of 1 and 10 mM AsA slightly accelerated cell division, however without reaching statistical significance. Cell treatment with 100 mM AsA prolonged generation time significantly. Each point represents mean value ± SD of 4 individual samples. Statistical differences are indicated as $p < 0.05$ *, 0.01 **, 0.001 ***.

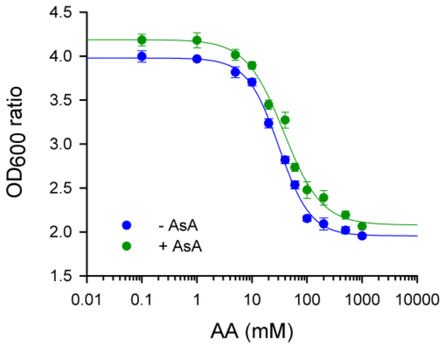

Figure S4. Determination of IC_{50} upon AA exposure with and w/o AsA pre-treatment. The half critical value of AA causing growth retardation of 50 % cells is 30.8 mM. However, AsA (10 mM) pre-treatment increased the amount of AA required for growth retardation to 37.8 mM referring the positive effect of AsA against AA-induced toxicity.

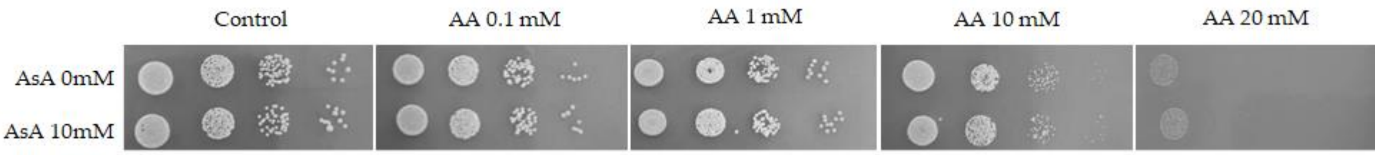

Figure S5. Spot test upon AA addition with and w/o AsA pre-treatment. Cells from the overnight culture un- and pre-treated with AsA were serially diluted and 10, 100, 1000, 10 000 cells were plated on plates containing indicated amount of AA. After 2 – 3 days of incubation at 30 °C, formed colonies or spots were compared. Increasing AA concentration reduced the ability of cells to grow, AsA pre-treatment, in this particular type of experiment, was not able to alleviate AA toxicity.

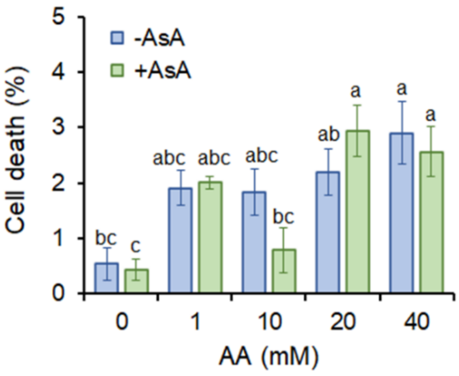

Figure S6. Cell viability upon AA addition with and w/o AsA pre-treatment. Cell viability is represented as percentual portion of dead to living cells determined by methylene blue staining. Each bar represents mean value ± SD of 4 individual samples. Statistical significance is determined by Duncan's post-hoc test, different letters above bars indicate statistical difference at $p < 0.05$.

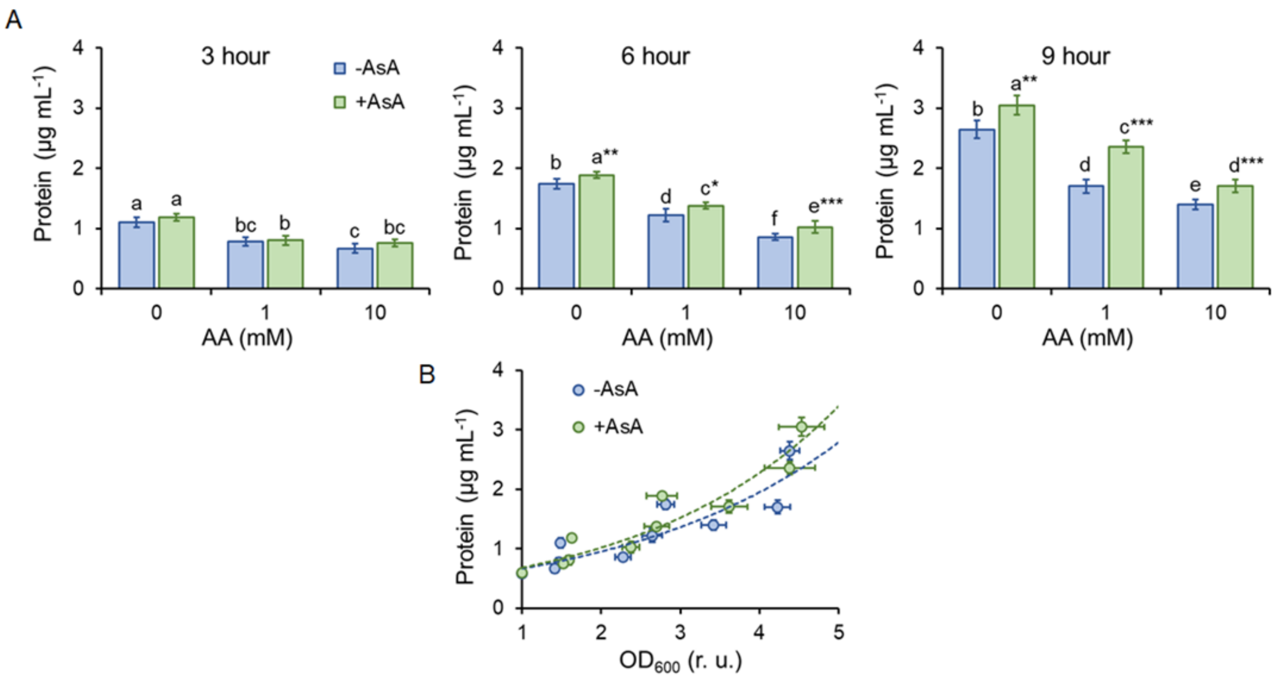

Figure S7. Protein content. Protein content was determined by Bradford assay. (A) Protein content decreased with increasing AA concentration, AsA pre-treatment elevated amount of protein (B) Protein content was proportional to OD_{600}. Each bar or point represents mean value ± SD of 4 individual samples. Statistical significance is determined by Duncan's post-hoc test, different letters above bars indicate statistical difference. Statistical differences are indicated as $p < 0.05$ *, 0.01 **, 0.001 ***.

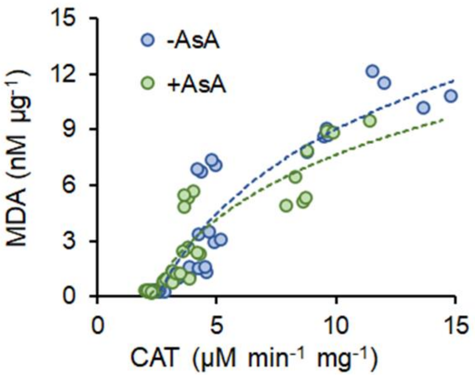

Figure S8. CAT/MDA ratio upon AA addition with and w/o AsA pre-treatment. Increasing MDA content enhanced CAT activity referring to increasing oxidative stress that induced more extensive antioxidant protection by elevated CAT activity. Each point represents mean value of 4 individual samples.
